# A long-term field experiment of soil transplantation demonstrating the role of contemporary geographic separation in shaping soil microbial community structure

**DOI:** 10.1002/ece3.1006

**Published:** 2014-03-06

**Authors:** Bo Sun, Feng Wang, Yuji Jiang, Yun Li, Zhixin Dong, Zhongpei Li, Xue-Xian Zhang

**Affiliations:** 1 State Key Laboratory of Soil and Sustainable Agriculture, Institute of Soil Science, Chinese Academy of Sciences71 East Beijing Road, Nanjing, 210008, China; 2 University of the Chinese Academy of SciencesBeijing, 100049, China; 3 College of Resource & Environment, Sichuan Agricultural UniversityChengdu, 611130, China; 4 Institute of Mountain Hazards and Environment, Chinese Academy of ScienceChengdu, 610041, China; 5 Institute of Natural and Mathematical Sciences, Massey University at AlbanyAuckland, 0745, New Zealand

**Keywords:** 454 pyrosequencing, contemporary disturbance, historical contingency, microbial biogeography, nitrogen cycling, soil transplantation

## Abstract

The spatial patterns of microbial communities are largely determined by the combined effects of historical contingencies and contemporary environmental disturbances, but their relative importance remains poorly understood. Empirical biogeographic data currently available are mostly based on the traditional method of observational survey, which typically involves comparing indigenous microbial communities across spatial scales. Here, we report a long-term soil transplantation experiment, whereby the same two soils (red Acrisol and purple Cambisol from Yingtan) were placed into two geographic locations of ∼1000 km apart (i.e., Yingtan in the mid-subtropical region and Fengqiu in warm-temperate region; both located in China). Twenty years after the transplantation, the resulting soil microbial communities were subject to high-throughput 454 pyrosequencing analysis of 16S and 18S rRNA genes. Additionally, bacteria and archaea involved in nitrogen cycling were estimated using clone library analysis of four genes: archaeal *amoA*, bacterial *amoA*,*nirK,* and *nifH*. Data of subsequent phylogenetic analysis show that bacteria, fungi, and other microbial eukaryotes, as well as the nitrogen cycling genes, are grouped primarily by the factor of geographic location rather than soil type. Moreover, a shift of microbial communities toward those in local soil (i.e., Chao soil in Fengqiu) has been observed. The results thus suggest that the historical effects persistent in the soil microbial communities can be largely erased by contemporary disturbance within a short period of 20 years, implicating weak effects of historical contingencies on the structure and composition of microbial communities in the soil.

## Introduction

The recognition that microorganisms display spatial biogeographic patterns has stimulated intense research interests concerning the underlying ecological and evolutionary mechanisms (Green and Bohannan 2006; Prosser et al. 2007; Ramette and Tiedje 2007; Maron et al. 2011; Hanson et al. 2012; Nemergut et al. 2013). The classic view of microbial biogeography – everything is everywhere, but the environment selects – highlights the potential unlimited dispersal capabilities of microorganisms and predicts that microbial community structure is predominantly determined by local environmental conditions (Baas-Becking 1934). However, it has been argued that everything is not really everywhere (Cho and Tiedje 2000; Green et al. 2004; Rout and Callaway 2012); past evolutionary and ecological events, including geographic separation, make significant contributions to the current assemblages of microbial communities (Martiny et al. 2006). Consequently, the spatial pattern of microorganisms has been hypothesized to be a result of interaction between two major factors, that is, historical contingencies and contemporary environmental disturbances (Martiny et al. 2006). However, their combined effects or relative contribution toward microbial assembling across spatial scales is currently poorly understood.

For microorganisms dwelling in soil, their community composition and activity are known to be strongly influenced by historical factors such as geographic location and soil type, as well as contemporary disturbances, which include transient change in soil pH, water content, minerals, organic manures, and tillage (Sayer et al. 2013; Sullivan et al. 2013). The spatial distribution of soil microbial communities has been well documented in the literature, including two recent large-scale surveys of bacterial populations in France and Great Britain (Griffiths et al. 2011; Maron et al. 2011; Ranjard et al. 2013). However, there is a lack of congruence with regard to the relative importance of historical versus environmental factors (for reviews, see (Martiny et al. 2006; Hanson et al. 2012)). Life history was identified to be the major driver of bacterial communities in soils over distances from m to 100 km in Australia and in soils across a regional scale of ∼1000 km in China (Ge et al. 2008; Bissett et al. 2010). In the contrast, Kuang et al. (2013) have shown that the bacterial communities in acidic mine drainage ecosystem were primarily determined by contemporary environmental variables rather than historical contingencies. A strong influence of local environment was reported on the composition of arbuscular mycorrhizal communities at the landscape scale (Hazard et al. 2013). Moreover, a number of studies have highlighted the roles of edaphic parameters, particularly soil pH, in explaining bacterial biogeography in the soil (Fierer and Jackson 2006; Lauber et al. 2009; Bru et al. 2011; Griffiths et al. 2011). Together, inconsistent results were obtained from different studies, and they were likely attributable to the related spatial scales or the specific types of microorganisms under investigation (Martiny et al. 2006; Bissett et al. 2010; Ragon et al. 2012). Clearly, more studies are needed in order to establish the common themes with regard to the relative importance of historical and contemporary factors.

In terms of methodology, data currently available are mostly based on microbiological surveys of soils under various geographic locations, which rely on robust statistical analysis to dissociate the combined effects of historical contingencies and contemporary disturbances. Alternatively, questions pertaining to the relative importance of history and environment can be powerfully addressed through studies of soil transplantation, whereby different types of soils are transferred to different geographic locations, followed by comparative analysis of microbial communities over a short or long period of time (Waldrop and Firestone 2006; Rinnan et al. 2007; Lazzaro et al. 2011). Of particular importance is that the initial microbial communities for each soil type have shared exactly the same evolutionary and ecological history in the past thousands, if not millions, of years. If history is the predominant determining factor, microbial communities in the same type of soil should remain closely related in different geographic locations after the transplantation. Conversely, if environment plays a pivotal role in driving microbial diversification, the effects of historical contingencies can be potentially erased within a reasonable short period of time, resulting in close relatedness of soil microbial communities in the same geographic location. In a previous report, we have performed a short-term reciprocal transplantation of three types of soils in three geographic locations; and the results showed that, at 4.5 years after soil transplantation, bacterial communities for each type of soil remained closely related at different sites (Sun et al. 2013). This initial finding was not surprising as soil is highly robust (Girvan et al. 2005; Rinnan et al. 2007). Few years can be considered very short, when compared with the thousands or millions of years that a soil formation process would have normally taken from parental materials (Huggett 1998).

Here, we describe a long-term soil transplantation experiment to assess the potential role of contemporary geographic separation in driving diversification of soil microbial communities. To this end, red soil (Acrisol) and purple soil (Cambisol) from a mid-subtropical region were placed into two geographic locations of ∼1000 km apart in China. Twenty years after the transplantation, the resulting microbial communities were compared using cultivation-independent molecular techniques, which were based on sequence analysis of 16S and 18S rRNA genes and clone library analysis of genes involved in nitrogen cycling. A focus on both taxa and functional traits was considered crucial for a better understanding of microbial biogeography (Green et al. 2008; Maron et al. 2011), and moreover, N-cycling microbial communities have become the models of investigation because of their importance in agriculture and environmental protection (Philippot et al. 2009; Bru et al. 2011; Martiny et al. 2011). Therefore, in addition to the general bacterial and eukaryotic microbial communities (e.g., fungi, protists, algae, and nematodes), specific functional groups (i.e., bacteria and archaea involved in nitrification, denitrification, and nitrogen fixation) were also included in this study. Finally, immigration and extinction are two important processes for biological diversity (Martiny et al. 2006; Fukami et al. 2007; Nemergut et al. 2013), but such events can rarely be recorded in microbial communities, partially due to the lack of high-throughput microbial identification techniques. Thus, the 454 pyrosequencing technique was employed to detect the potential emergence and disappearance of microbial taxa occurred over the course of soil transplantation.

## Materials and Methods

### Site description and soil sampling

The soil transplant experiment was set up in September of 1988 to study the long-term effects of two climate regimes on decomposition of soil organic matter and the associated microorganisms. The work involved two Agro-ecological Experimental Stations administrated by the Chinese Ecosystem Research Network (CERN), which are located in two climate regions with a distance of ∼1000 km: Yingtan (YT) in humid mid-subtropical monsoon climate region with an average annual temperature of 17.6°C; Fengqiu (FQ) in semihumid warm-temperate monsoon climate region with an average annual temperature of 13.9°C (Fig. S1). Two typical agricultural soils of the subtropical region (red soil and purple soil) were placed in parallel into the two experimental sites of YT and FQ. Specifically, each experimental site contained two cell units (2 m apart) for each of the two soils; each cell unit was built up with cement mortar brick wall (10 cm in thickness) with a final volume of 0.5 m^3^ (1 m length, 1 m width, and 0.5 m depth). One layer (3 cm in thickness) of quartz sands was placed at the bottom, and the units were 15 cm above the well-drained surrounding soils. The purple and red soils were taken from their original location at a depth of 0.5 m by three layers (20 cm at the top, 20 cm in the middle, and 10 cm at the bottom); the soil samples from each layer were mixed and put into plastic bags. After the bagged soils were transported to the two new experimental sites, they were placed into the pre prepared cell units according to their original order.

The transplanted red and purple soils had been subjected to the same agricultural practices in terms of crop rotation and fertilization. Specifically, the soils in FQ were cultivated at maize–wheat rotation with annual N-P-K, 250-75-60 kg·ha^−1^, whereas soils in YT were grown at peanut–rape rotation with annual N-P-K, 120-75-60 kg·ha^−1^. In September 2009 – twenty years after transplantation – soil samples were taken from the top layer (0–15 cm) using a stainless steel cylinder (3 cm inner diameter). To analyze the microbial communities, for each of two soils (purple and red) in two locations (YT and FQ), three bulked soil samples were collected after dividing each plot into three equal subplots, giving rise to three replicates for each. Additionally, the local Chao soil in FQ was sampled in three replicates at a distance of ∼20 m from the experimental plots, acting as a control of the local microbial community. Of note, the red soil (Acrisol) and purple soil (Cambisol) with silty clay texture are derived from Quaternary red clay and purple sandstone, respectively, whereas the Chao soil (Cambisol) with sandy loam texture is derived from alluvial sediments. The obtained soil samples (totally five, with three replicates for each) were stored at 4°C for microbial analysis, and some were air-dried and sieved (2 mm) for physiochemical analysis.

### X-ray diffraction analysis

Standard methods were used to characterize the soil physicochemical properties such as pH, soil organic matter and total nitrogen, and total and available phosphate and potassium. Soil samples for the X-ray diffraction (XRD) analysis were prepared as previously described (Moore and Reynolds 1997; Laird 1999). Briefly, soil samples were first treated with 0.1 mol·L^−1^ HCl to remove free carbonates and then subject to wet oxidation with 30% H_2_O_2_ in order to remove the organic matter. Next, 0.5 mol·L^−1^ NaOH was used to adjust pH of the suspension to 7.3, and the fraction of <2 *μ*m was separated by sedimentation. To further purify the clay, free ionic compounds were removed by adding sodium dithionate, sodium citrate, and sodium bicarbonate at the final concentration of 1.5 mol·L^−1^, 0.3 mol·L^−1^, and 1.0 mol·L^−1^, respectively. Magnesium-saturated samples were prepared and oriented on glass slides by the paste method (Theissen and Harward 1962).

X-ray diffraction was performed on a Japan Rigaku D/max-3C X-ray diffractometer with graphite-monochromatized Cu Ka radiation (*λ *= 0.154178 nm) following the manufacturer's recommendation. Standard minerals were used to develop relationships between XRD integrated intensity ratios and weight fraction ratios of mineral contents (Moore and Reynolds 1997).

### Soil DNA preparation and 16 rDNA DGGE

Total DNA was extracted from 0.5 g soil using the FastDNA SPIN Kit for soil from MP Biomedicals (Santa Ana, CA) following the manufacturer's instructions. All oligonucleotide primers used in this study are listed in Table S3. For 16S rDNA denaturing gradient gel electrophoresis (DGGE) analysis, primers PRBA338F with GC clamp and PRUN518R were used to amplify the variable V3 region of the bacterial 16S rRNA (Muyzer et al. 1993). The resultant PCR products were subject to polyacrylamide gel electrophoresis with a denaturing gradient of 35–60%. Ribosomal genotypes and their relative abundance were estimated using band position and intensity, which were analyzed with the help of the Quantity One program (Bio-Rad Laboratories, Hercules, CA).

### RFLP analysis and DNA sequencing of nitrogen cycling genes

Four genes involved in nitrogen cycling (archaeal *amoA*, bacterial *amoA*,*nifH,* and *nirK*) were amplified from the total DNA extracted from the soil using four pairs of primers that are listed in Table S3 (Rotthauwe et al. 1997; Braker et al. 1998; Rosch et al. 2002; Francise 1997). The PCR products were cloned into a TA-cloning vector pMD18-T (TaKaRa, Dalian, China) after purification using the UltraClean 15 purification kit (Mo Bio, Inc., Solana Beach, CA). About 100 transformants were randomly picked up and subjected to restriction analysis with two enzymes (*Hha*I and *Sau*3AI for *amoA* and *nirK*;*Hha*I and *Afa*I for *nifH*). Gene clones with unique restriction fragment length polymorphism (RFLP) profiles were then subjected to DNA sequencing at Majorbio (Shanghai, China). A total of 43, 31, 27, and 49 DNA sequences were successfully obtained for archaeal *amoA*, bacterial *amoA*,*nifH,* and *nirK,* respectively. DNA sequences were aligned using the Clustal W program in BioEdit package (Hall 1999), and phylogenetic analysis was performed using the MEGA version 5.0 (Tamura et al. 2011). The radiated neighbor-joining tree was constructed using the nucleotide sequence distance measurement Poisson correction model. Genetic structure maps of four nitrogen cycling genes were generated by combining the radiation tree and relative abundance of clones in each RFLP pattern.

## 454 pyrosequencing of 16S or 18S rDNAs

The 16S (V1–V3 region) or 18S (V4 region) rDNAs were amplified using the two pairs of primers listed in Table S3, and subsequent 454 pyrosequencing was performed by the Shanghai Majorbio Bio-Pharm Biotechnology Co. (Shanghai, China) using the Genome Sequencer FLX Titanium instrument (Roche, Nutley, NJ). Low-quality sequences were removed through several quality filters as previously described (Huse et al. 2007). Specifically, the following reads were omitted from further analyses: reads lacking the barcode and forward primer, reads with ambiguous nucleotides, reads of <150 nucleotides, reads with more than two erroneous bases from the forward primer, and reads containing homopolymer runs greater than six bases. The remaining sequences were aligned against Mothur's Silva 106 reference database and were trimmed to cover the same region (Pruesse et al. 2007; Schloss et al. 2009). Sequences that did not align correctly were then removed from the dataset. A total of 26,200 16S rDNA and 27,007 18S rDNA sequences were obtained with an average length of 433 bp and 406 bp, respectively. Taxonomic identity was based on a query sequence against the reference sequences in the Silva 106 database. The level of the most resolved taxon shared by at least 80% of the reference sequences was classified. The average neighbor clustering algorithm was utilized for operational taxonomic unit (OTU)-based analyses to estimate the species richness. Rarefaction curves, Shannon diversity index, and two richness estimators (Chao1 and ACE) were calculated using Mothur (Schloss et al. 2009).

## Statistical analysis

Hierarchical cluster analysis, an agglomerative method that creates groups from multivariate data using a dissimilarity matrix, was used to categorize samples based on OTUs data matrix generated from 454 pyrosequencing and RFLP analysis (Everitt and Hothorn 2011). Hierarchical cluster was created with group average linkage using Bray–Curtis distance measurement and performed using the “Vegan” package in R statistical programming version 2.13.2 (R-Development-Core-Team 2008; Oksanen et al. 2011). One-way analysis of variance (ANOVA) followed by Duncan's test was used to determine significant differences in physicochemical variables between different treatments and performed using version 16.0 SPSS software (SPSS Inc., Chicago, IL).

Canonical correspondence analysis (CCA) was supported by the gradient length in detrended correspondence analysis (DCA). Manual forward selection was then used to identify variance of species assemblages and environmental factors, with variables being rejected by the Monte Carlo permutation test (*P *>* *0.05) and the calculated variance inflation factors (VIF < 20). CCA was performed using the vegan package (v.1.17-9) in R statistical programming version 2.13.2, and the results were confirmed using CANOCO 4.5 for Windows (Biometris – Plant Research International, Wageningen, The Netherlands).

## Results

Soil samples were taken from the same red and purple soils that were placed into two geographic locations in China for 20 years: Yingtan in the south and Fengqiu in the north (Fig. S1). Also included into this work is the local Chao soil in the experimental site of Fengqiu. A total of five soil samples were subjected to the chemical, physical, and microbiological analyses described below.

### Physicochemical properties of soils after long-term transplantation

**Figure 1:**
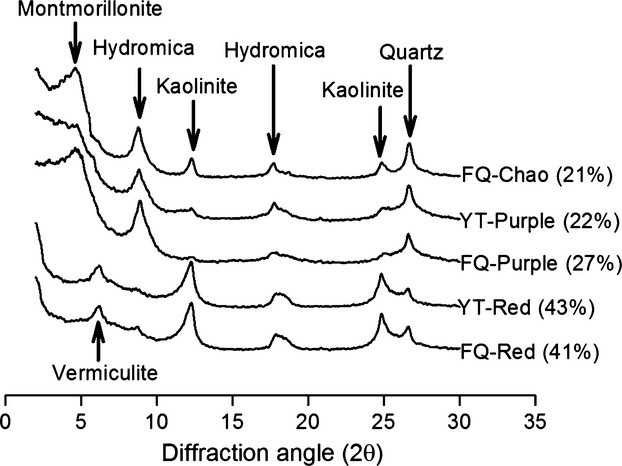
Comparison of the X-ray diffraction patterns of the five soil samples. Positions of the seven major clay minerals are indicated with arrows and number in parenthesis denotes clay content (%) for each of the five soils. Soil samples are designated by location (FQ, Fengqiu; YT, Yingtan) and soil type (Chao, purple, or red soil).

Twenty years after the soil transplantation, both red and purple soils at the two geographic locations have remained their original appearance (e.g., the colors), suggesting negligible changes in clay and mineral content of the soils. To confirm this, the five soil samples were subjected to XRD analysis and the results are shown in Figure 1. Consistent with our prediction, similar XRD profiles were obtained for the two red soils and separately for the two purple soils. The Chao soil was a brown-colored local soil in Fengqiu, and it has the lowest clay content between the five soils analyzed. The most abundant clay minerals are kaolinite (65%) for red soil, hydromica (50–57%) for purple soil, and montmorillonite (39%) for Chao soil, regardless of their locations.

It is interesting to note some significant changes in soil properties, soil pH in particular (Table 1). The original pH at the time 0 was 5.15 for red soil and 7.16 for purple soil. After 20 years, the red soil has remained acidic in Yingtan (4.99 ± 0.03) but becomes lightly alkaline in Fengqiu (7.56 ± 0.54), whereas the purple soil has been acidified in Yingtan (5.80 ± 0.41) and slightly alkalized in Fengqiu (7.40 ± 0.06). Of note is that the local soil surrounding the experimental plot in Fengqiu (i.e., the Chao soil) has a pH of 8.02 ± 0.23. Therefore, the observed increase in pH for soils transferred to this site, and consistently the decrease in pH for purple soil in Yingtan, is likely attributable to the effects mediated by minerals in the underground waters. Other noticeable changes include higher soil organic carbon (SOC) and total nitrogen for red soil than for purple soil in Yingtan, lower total phosphate of soils in Yingtan than soils in Fengqiu (Table 1). Taken together, the long-term soil transplantation has resulted in significant changes in the chemical characteristics of the soils; however, clay compositions of the soils, which are determined by the parental materials, remained unaltered.

**Table 1:** Physicochemical properties of the soils involved in this work.

Soil samples^1^	pH	Water content (%)	SOC (g·kg^−1^)	Total N (g·kg^−1^)	Total P (g·kg^−1^)	Total K (g·kg^−1^)
FQ-Chao	8.02 ± 0.23 (a)	11.6 ± 0.6 (c)	6.23 ± 0.13 (c)	0.61 ± 0.01 (d)	0.90 ± 0.17 (abc)	18.4 ± 0.40 (a)
FQ-purple	7.40 ± 0.06 (b)	14.7 ± 0.2 (b)	8.00 ± 0.17 (b)	0.96 ± 0.04 (ab)	1.12 ± 0.07 (a)	18.2 ± 0.53 (a)
FQ-red	7.56 ± 0.54 (ab)	14.8 ± 0.4 (b)	8.08 ± 0.07 (b)	0.82 ± 0.09 (bc)	1.10 ± 0.08 (ab)	9.7 ± 0.04 (c)
YT-purple	5.80 ± 0.41 (c)	18.4 ± 0.5 (a)	6.39 ± 0.31 (c)	0.79 ± 0.03 (c)	0.62 ± 0.03 (c)	16.4 ± 1.00 (b)
YT-red	4.99 ± 0.03 (d)	19.6 ± 0.4 (a)	9.91 ± 0.13 (a)	1.03 ± 0.06 (a)	0.83 ± 0.02 (bc)	9.1 ± 0.14 (c)

1 Data are means and standard errors of three within plot replicates. Results of one-way ANOVA (Duncan) are shown in parenthesis with values identified by different letters being significantly different (*P *<* *0.05). When the soils were transplanted, their pH, soil organic carbon (SOC), and total N were measured as 5.15, 7.27, and 0.79, respectively, for red soil, 7.16, 6.02, and 0.79, respectively, for purple soil.

### Phylogenetic diversity of the soil-dwelling bacteria

**Figure 2:**
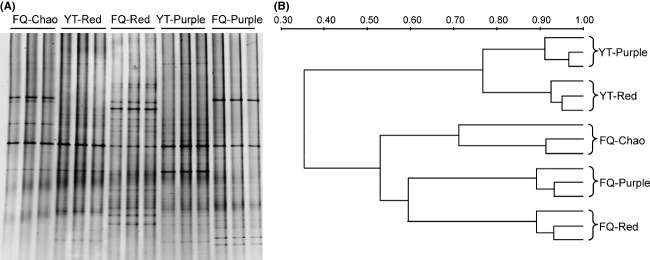
Photograph of the denaturing gradient gel electrophoresis (DGGE) analysis of the soil-dwelling bacterial communities (A) and the corresponding phylogenic relationships (B). DGGE analysis was performed by amplifying the V3 region of the 16S rRNA genes, and the dendrogram was generated using the cluster analysis (UPGMA) of the calculated Dice index.

To elucidate the community structure of bacteria in the soils, the well-established cultivation-independent technique of DGGE analysis was firstly employed and analysis of the 16S rRNA (V3 region) was performed with total DNAs from three soil samples (replicates) for each of the five treatments. The results presented in Figure 2 clearly showed that the bacterial communities were grouped primarily based on their geographic locations rather than the soil types. Bacterial communities of the red and purple soils in Fengqiu were more closely related to that of Chao soil from the same location, than the communities of the same types of soils located in Yingtan. The data provide a strong evidence of converged local adaptation of bacterial communities, with geographic location as the primary driving factor. Interestingly, difference in bacterial communities between the two transferred soils in Fengqiu is larger than that in their local region of Yingtan (Fig. 2, at the similarity level of 59% in Fengqiu versus 77% in Yingtan).

Next, we sought to determine the microbial composition using the high-throughput technique of 454 pyrosequencing. Given that the three soil samples for each treatment displayed the similar 16S rDNA DGGE profiles (Fig. 2), they were equally mixed for total DNA preparation and subsequent 454 pyrosequencing in order to reduce the experimental cost. The 454 pyrosequencing was performed using primers of 8f and 533r, which targets the V1–V3 region of the 16S rRNA. As summarized in Table 2 (Lu et al. 2012), an average of 5240 high-quality sequences were obtained for the five soils, which is sufficient to reflect the “species” distribution patterns of the bacterial communities on the basis of the detected dominant types (the rarefaction curves are shown in Fig. S3A). About 1500 OTUs were assigned at the level of 97% sequence similarity (Table 2). It appears that the local soils have higher levels of bacterial diversity than the foreign soils according to the estimated results of both species richness (Ace and Chao I indices) and evenness (Shannon index): in Table 2, FQ-red and FQ-purple soils versus the local soil of FQ-Chao; YT-purple versus the local soil of YT-red.

**Table 2:** Richness estimates and Shannon diversity indices for bacterial and eukaryotic taxa defined at a 97% similarity cutoff.

Samples	Bacterial					Eukaryotic				
	Total reads	OTUs	Ace	Chao I	Shannon	Total reads	OTUs	Ace	Chao I	Shannon
FQ-Chao	5392	1584	2702	2971	6.808	4158	732	1187	1218	5.527
FQ-purple	5598	1524	2567	2671	6.592	4492	760	1219	1171	5.616
FQ-red	5077	1312	2151	2194	6.453	4777	739	1138	1152	5.537
YT-purple	5337	1526	2581	2740	6.665	8374	1036	1569	1586	5.549
YT-red	4796	1498	2629	2658	6.706	5206	645	1079	1138	4.783

FQ, Fengqiu; OTUs, operational taxonomic units; YT, Yingtan.

**Figure 3:**
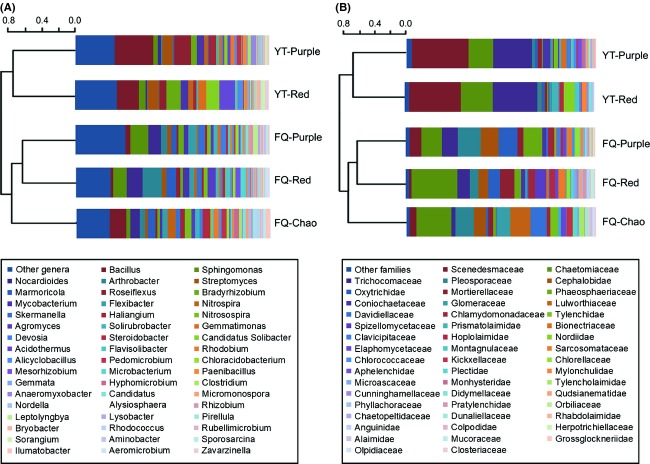
Cluster analysis of operational taxonomic units (OTUs) obtained from the 454 pyrosequencing of the 16S rDNA (A) and 18S rDNA (B) of soil-dwelling microbial communities. OTUs are defined by a 97% similarity cutoff and the relative abundance of the identified genus is shown by different colors. Soil samples are designated by location (FQ, Fengqiu; YT, Yingtan) and soil type (Chao, purple, or red soil). Proportion of all minor taxa (i.e., <40 reads for 16S rDNA and 14 reads for 18S rDNA in all five samples) is shown as other genera or other families, respectively.

The OTUs were then subjected to the Bray–Curtis cluster analysis. Results presented in Figure 3 are consistent with the conclusion drawn from the DGGE analysis described above that the bacterial communities are grouped primarily by the factor of geographic location, and not soil type. Interestingly, *Bacillus* is the most dominant genus in the red and purple soils in Yingtan, but it was much less commonly found for the two soils in Fengqiu. In the contrast, several ubiquitous taxa such as *Sphingomonas*,*Nocardioides, Arthrobacter*, and *Marmoricola* were more predominantly detected in the two soils located in Fengqiu, than the two soils from Yingtan (Table S1). It is interesting (but not surprising) that the acidophilic genus *Acidothermus* was predominantly found in the two soils from Yingtan (∼100 sequences), and it was not detected in the red soil and purple soil from Fengqiu (Table S1). This result is correlated with the relative low pH of soils in Yingtan (Table 1), thus highlighting the importance of soil pH in selecting for acidophilic bacteria. It is also worthy mentioning that taxa such as *Streptomyces* and *Bradyrhizobium* were ubiquitously found in all five soils at similar levels, suggesting that they are not responsive to soil transplantation. Finally, CCA analysis was performed, and the results showed that soil parameters such as pH, water content, total N, and SOC contribute to 11.2%, 9.3%, 7.3%, and 6.0% of the variation, respectively (Fig. S2).

### 454 pyrosequencing of the eukaryotic microbes in the soil

To get a glimpse of eukaryotic microbial communities in the soils, total DNAs from the five soils were subject to 454 pyrosequencing of 18S rDNA using a pair of primers 3NDf and V4_euk_R2 (Brate et al. 2010). After low-quality sequences and those of plant and insects were removed from the datasets, an average of 5401 sequence reads and 782 OTUs with a 97% cutoff were detected for each soil sample. The results shown in Table S2 indicate that dematiaceous fungi belonging to the *Chaetomiaceae* family are the most dominant eukaryotic microbes found in all five soils (ranging from 12% to 28%). Another ubiquitous fungal family present at relative lower frequencies was *Trichocomaceae*, which were more predominantly found in soils from Yingtan than the three soils located in Fengqiu. *Trichocomaceae* contains many species known to live in soils saprotrophically on decomposing plant residues (Hibbett et al. 2007). Intriguingly, other fungi such as *Lulworthiaceae* were found only in soils from Fengqiu.

Ciliated protozoans (family *Oxytrichidae*) and nematodes in the *Cephalobidae* family were ubiquitously found in all five soils with relatively high frequency in the two translocated soils in Fengqiu. Notably, algae in the *Scenedesmaceae* family and the *Prismatolaimidae* nematodes were detected at high frequencies in soils in Yingtan, but little or none in soils from Fengqiu. While the precise ecological factors that determine these biased distributions are unknown, they are likely attributable to the lower pH and/or higher soil water contents for soils in Yingtan (Table 1). In contrast to the clear location-associated distributions described above, the effects of soil type are not noticeable for the detected dominant taxa (Table S2). Finally, cluster analysis of the eukaryotic OTUs showed that eukaryotic microorganisms in soils of the five treatments were also grouped by geographic locations, which are very similar to the bacterial communities (Fig. 3B). Notably, when the analysis was performed with each of four types of microbial eukaryotes (i.e., fungus, alga, nematode, and protist), close relatedness was found for communities in the same geographic location (Fig. S4), suggesting similar effects for all eukaryotic microbial taxa.

### Effects on diversification of functional genes involved in nitrogen cycling

Finally, we aimed to elucidate the effects of soil transplantation on functional diversity of microorganisms that are of particular importance in agriculture. To this end, we have focused on the microbial communities involved in nitrogen cycling in soil. Nitrogen is an essential and often limiting element for plant growth and the use of nitrogen fertilizers has been a key agricultural input (Frink et al. 1999). However, overuse or misuse of nitrogen fertilizers can cause serious environmental and health problems (Tilman et al. 2002; Conley et al. 2009). The analysis was performed with four functional genes, that is, bacterial *amoA*, archaeal *amoA*,*nirK,* and *nifH*. They encode three enzymes – ammonia monooxygenase subunit A (AmoA), copper-containing nitrite reductase (NirK), and nitrogenase iron protein (NifH) – which are involved in the process of nitrification, denitrification, and nitrogen fixation, respectively (He et al. 2010; Bru et al. 2011).

**Figure 4:**
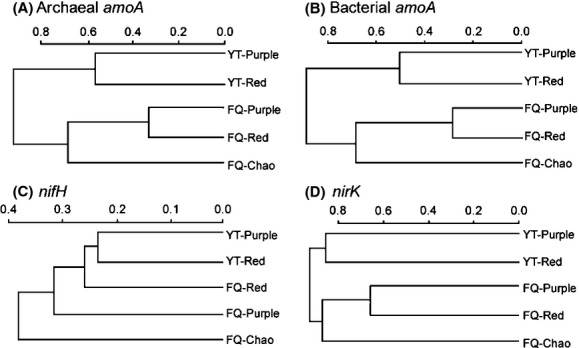
Phylogenetic relationships of bacterial communities based on restriction fragment length polymorphism (RFLP) analysis of archaeal *amoA* (A), bacterial *amoA* (B),*nifH* (C), and *nirK* (D) genes. The dendrograms were generated using hierarchical cluster analysis based on group-average linking of Bray–Curtis similarities calculated from the obtained RFLP data. Soil samples are designated by location (FQ, Fengqiu; YT, Yingtan) and soil type (Chao, purple, or red soil).

Clone libraries were constructed from total DNAs of the five soil samples for each of the four functional genes. A total of 486 archaeal *amoA*, 488 bacterial *amoA*, 333 *nifH,* and 255 *nirK* clones were randomly picked and were then subjected to RFLP analysis. The RFLP analysis with two restriction enzymes produced approximate 10 or 20 OTUs (Table 3). Significantly, results of cluster analysis shown in Figure 4 indicate that the denitrifying communities (*nirK*) and the nitrifying communities (*amoA* for both archaea and bacteria) are all closely related to each other for soils from the same geographic location. The phylogenetic relationships of *nifH* genes are in agreement in general with those of *nirK* and *amoA* (Fig. 4C). While the *nifH* genes in red and purple soils in Yingtan are grouped together, the *nifH* gene pools in the three soils located in Fengqiu display a relatively high degree of divergence. Given that the three soils in Fengqiu have been subjected to the same field management, the divergence of *nifH* at this site suggests a strong effect of soil type on the nitrogen fixing communities under the climate conditions of Fengqiu.

**Table 3:** A summary of the number of clones and unique restriction profiles for restriction fragment length polymorphism analysis of genes involved in nitrogen cycling.

Samples	Archaeal *amoA*		Bacterial *amoA*		*nifH*		*nirK*	
	Clones	OTUs	Clones	OTUs	Clones	OTUs	Clones	OTUs
FQ-Chao	98	21	97	18	66	9	55	42
FQ-purple	95	20	97	15	59	13	41	18
FQ-red	98	13	100	15	68	9	54	35
YT-purple	99	16	96	13	66	11	70	49
YT-red	96	18	98	13	74	12	35	25

FQ, Fengqiu; OTUs, operational taxonomic units; YT, Yingtan.

**Figure 5:**
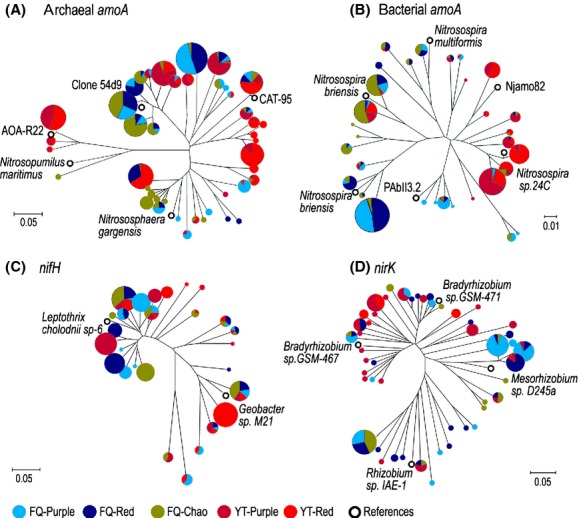
Neighbor-joining trees generated from data of DNA sequences of the archaeal *amoA* (A), bacterial *amoA* (B),*nifH* (C), and *nirK* (D) genes, respectively. Only one representative clone from each of the unique operational taxonomic units identified by restriction fragment length polymorphism analysis was subjected to DNA sequencing, and their relative abundance was shown by the sizes of the cycles. Soil samples are designated by location (FQ, Fengqiu; YT, Yingtan) and soil type (Chao, purple, or red soil). The following reference sequences were obtained from the GenBank database (accession numbers in parentheses): Clone 54d9 (AJ627422), AOA-R22 (FJ517351) *Nitrosopumilus maritimus* (DQ085098), *Nitrososphaera gargensis* (EU281321), CAT-95 (GQ481089), *Nitrosospira multiformis*, (X9082), *Nitrosospira briensis* (AY123821), *Nitrosospira briensis* (U76553), PAbII3.2, (AJ388582), *Nitrosospira* sp. 24C (AJ298685), Njamo82 (AF356515), *Leptothrix cholodnii* sp-6 (CP001013), *Geobacter* sp. M21 (CP001661), *Bradyrhizobium* sp. GSM-467 (FN600568), *Rhizobium* sp. IAE-1 (HM060300), *Mesorhizobium* sp. D245a (AB480470), *Bradyrhizobium* sp. GSM-471 (FN600571).

Next, we sequenced the unique OTUs identified in the PCR-RFLP analysis. The resulting neighbor-joining trees are shown in Figure 5 with the five soils being represented by different colors. A cursory examination of the trees shows that most genotypes from the same location are clustered together (the two soils in Yingtan are identified using red colors, which are distinguishable from soils in Fengqiu). In some cases, the same genotypes were found in all five soils, for example, the archaeal *amoA* genotype related to the reference sequence of CAT-95 (Fig. 5A). For archaeal *amoA*, soils in Fengqiu were dominated by members of the 54d9 cluster (Treusch et al. 2005), which was equivalent to the group 1.1b as previously identified by Ochsenreiter et al.(2003); however, soils in Yingtan belonged to the cluster with references sequences of CAT-95 or AOA-R22 (Ying et al. 2010; Navarrete et al. 2011). All bacterial *amoA* sequences were affiliated with the genus of *Nitrosospira*: most clones from Yingtan were grouped into cluster 10 and cluster 12 with *Nitrosospira*sp. 24C and Njamo82 as the respective reference sequences, whereas the clones from Fengqiu belonged to cluster 3 (*Nitrosospirabriensis* and *Nitrosospiramultiformis*, Fig. 5B), which are commonly found in many soils (Avrahami and Conrad 2003; He et al. 2007). The *nirK* sequences for denitrifying bacteria were categorized into three major groups (Fig. 5D): *Bradyrhizobium* (mostly isolated from soils in Yingtan), *Mesorhizobium,* and *Rhizobium* (which dominate the clones from Fengqiu). The *nifH* sequences were mainly affiliated with *β*-*Proteobacteria* (*Leptothrixcholodnii* SP-6) and *δ*-*Proteobacteria* (Geobacter sp. M21), with few sequences being classified into *Chlorobia* and *Clostridia* (data not shown). However, the *nifH* sequences did not show a clear separation by location, which has been observed for the *amoA* and *nirK* clones.

## Discussion

Data presented in this report show that microbial communities in red and purple soils were shifted toward to the local microbial communities in Chao soil, which occurred within 20 years after the two soils were transferred from a mid-subtropical region to a warm temperate region across a distance of ∼1000 km. Significantly, similar effects were observed for bacteria, fungi, and other microbial eukaryotes, as well as specific functional groups of N-cycling bacteria and archaea. The results provide a unique support to the Baas-Becking's idea that “the environment selects.” However, we are unable to claim that the effects were attributable to “everything is everywhere.” This open field experiment was set up to mimic the natural conditions where different types of soils coexist in the same climate region. Foreign microorganisms are allowed to enter into the soil system by many ways: from the top via air and rain/snow and from the bottom via ground water; however, not all the emigrating microorganisms can successfully invade the indigenous soil microbial communities, which are spatially structured. Given that microbes located nearby have adapted well to the local climate conditions, they would have a much higher chance than those from a long distance to invade the existing microbial communities of the transferred soils. Apart from species invasion, it is also likely that some preexisting common species would have changed in frequency as a result of adaptation to the local environmental conditions. These may partially explain that the resulting microbial communities in the transferred red soil and purple soils were closely related to the local Chao soil at the experimental site of Fengqiu.

The most important message from this study is that the effects of past evolutionary and ecological events on microbial community composition can be partially erased by contemporary disturbance within a short period of time, suggesting weak effects of historical contingencies on the current assembly of soil-dwelling microbial communities. This finding is in general agreement with previous reports that show stronger effects of contemporary environments than historical contingencies on microorganisms at both landscape and regional scale (Hazard et al. 2013; Kuang et al. 2013). Moreover, it should be noted that change in climate regimes is an important contemporary disturbance in this work (Jackson et al. 2009). Therefore, our results suggest that the soil microbial communities are more susceptible to climate change (and agricultural practices) than we would normally think (Chung et al. 2007; Zhou et al. 2012).

Structure of microbial communities is a historical product of diversification, immigration, and extinction (Fukami et al. 2007; Nemergut et al. 2013). In this work, the high-throughput 454 pyrosequencing technique has been used to elucidate the community composition of bacteria and microbial eukaryotes. The analysis provided a unique opportunity to detect the emergence and disappearance of microbial “species” over the course of soil transplantation. For example, bacteria in the genus of *Acidothermus* (Table S1) and fungi *Montagnulaceae* (Table S2) were commonly found in red soil located in its original region of Yingtan, but they were undetectable in red soil located in the new region of Fengqiu. On the contrary, bacteria such as *Skermanella*,*Agromyces,* and *Marmoricola* (Table S1) and fungi in the families of *Spizellomycetaceae* and *Sarcosomataceae* (Table S2) are present in abundance in red soil in Fengqiu, but not in its original region of Yingtan. Finally, it is interesting to note that organisms such as the *Trichocomaceae* fungi are abundant in both red and purple soils in Yingtan, but greatly reduced in the transferred red and purple soils in Fengiu and undetectable in the local Chao soil. This result suggests that this group of microorganisms is not favored in this particular warm temperate region regardless of which type of soil they reside, which may be caused by change in climate regimes and other geographic factors (Castro et al. 2010).

Crop rotation has significant effects on microbial communities in the soil (Sun et al. 2011; Murugan and Kumar 2013). Of particular note are leguminous plants that form nitrogen fixing nodules with certain group of Gram-negative bacteria (e.g., *Rhizobium*,*Mesorhizobium,* and *Bradyrhizobium*). In this work, change in crop rotation is considered as a factor of contemporary disturbance, and the work involved one leguminous plant (i.e., peanut), which had been grown in rotation with oil rape in the fields of Yingtan before and after the soil transplantation. Our data presented in Table S1 show that similar levels of *Bradyrhizobium* were observed in all five soils; *Rhizobium* and *Mesorhizobium* were present in soils from Fengqiu, but none or little were detected in soils from Yingtan. This result was initially surprising as peanut was expected to increase the frequencies of the rhizobial populations. However, it can be partially explained by the possibilities that certain rhizobial strains can grow saprophytically in the absence of host plants and that acidic soils in Yingtan may not be suitable for the rhizobial populations (Hirsch 1996; Zhang et al. 2001). Additionally, the microbiological methods based on 16S rRNA and NifH genes do not allow precise identification of the symbiotic bacteria associated with peanut.

Current studies of microbial biogeography have mostly focused on the phylogenetic distribution of microorganisms based on the analysis of ribosomal RNA genes, and specific functional groups of the communities have rarely been targeted (Green et al. 2008; Lüke et al. 2010; Bru et al. 2011). In a previous work by Lüke et al. (2010), methane-oxidizing bacteria (MOB) in rice paddy fields have been examined at a geographic scale of 10–20 km, and the data of comparative analysis of PmoA genes suggested that the observed site difference in MOBs was likely due to effects of historical contingencies in the form of soil types. Here, we analyzed functional genes involved in nitrogen fixation (*nifH*), nitrification (bacterial and archaeal *amoA*), and denitrification (*nirK*). Nitrogen cycling is a major biogeographic cycle that involves the interconversion of various nitrogen species (e.g., N_2_, NH_4_^+^, NO_3_^−^, NO_2_^−^, and NO), playing important roles in determining the productivities of various ecosystems (Jia and Conrad 2009; Bru et al. 2011). Interestingly, this work has revealed similar distribution patterns of nitrogen cycling genes as those observed for total bacteria and microbial eukaryotes, implicating the important roles of contemporary disturbances in nitrogen cycling in soil. The inconsistency between microbes of methane oxidizing and nitrogen cycling may reflect the dramatic difference in environments between paddy fields and upland soils. The paddy fields are subject to alternative flooding and drying, which may override the effects of other climate condition such as temperature. Moreover, the relatively larger geographic distance tested in this work (∼1000 km) is also expected to produce stronger effects than those involved in the previous study on MOBs (Martiny et al. 2006; Lüke et al. 2010).

The microbial communities were examined only at one time point in this work, and it is thus possible that the observed transition of microbial communities can take place even shorter than 20 years. In a separate large-scale soil transplantation experiment, which started later in 2005 and involved a reciprocal transfer of three agricultural soils (red soil, Chao soil, and black soil) in three climate regions of China (mid-subtropical, warm-temperate, and cold-temperate zones), we have monitored the dynamic changes in bacterial communities in bulk soils using 16S rDNA DGGE analysis during a period of 4.5 years (Sun et al. 2013). Results show that the soil microbial communities were still primarily grouped by soil type, not by geographic location as we observed in this study. Therefore, it likely took about 5–20 years for the detected shift of microbial community composition. Interestingly, Rinnan et al. (2007) examined the soil microbial communities in a subarctic health ecosystem following manipulation of climate conditions, and their results demonstrated that more than 10 years is needed for the production of significant responses.

Transplantation has long been used in geobotany to study the adaptation of plants or plant-associated pathogens to different environmental conditions (Scheepens et al. 2010). The technique offers a great advantage by eliminating the potential biases in maintaining the designed plant growth environments in the laboratory. The similar strategy of soil transplantation has recently drawn increasing attention for studies of microbial communities, in response to various environmental changes such as vegetation (Balser and Firestone 2005; Waldrop and Firestone 2006; Vanhala et al. 2011), temperature (Link et al. 2003; Zumsteg et al. 2013), and soil properties (Lazzaro et al. 2011). In these work, only a small amount of surface soils (∼10 kg) were transferred and they were placed either in sealed incubation vessels or pots buried in soil; moreover, microbial communities were monitored within a short period of time (<2 years). However, the soil transplantation experiments performed in this laboratory involved large-scale transplantation of soils of 0.5 m in depth, whereby the original structure of the soils was maintained and thus more suitable for long-term investigation into the effects of various geographic factors.

Taken together, data obtained from this long-term soil transplantation experiment complement the previous survey-based studies of microbial biogeography, implicating weak influences of historical contingencies on microbial spatial distribution. Furthermore, this work also demonstrates the usefulness of the transplantation technique in research on soil microbial community structure to assess the influences of geographic and soil parameters. Finally, it should be noted that another ongoing large-scale soil transplantation experiment setup in 2005 has seven replicate plots for each of the three soils in three geographic locations, and the microbial communities have been subjected to annual monitoring. It would thus be interesting to know whether or not the conclusions made from this study with one transplant plot per soil type will hold, and moreover, the detailed dynamic changes in microbial community over time.

## Data Accessibility

DNA sequences of nitrogen cycling genes are available with the GenBank Accession Numbers: KC773876–KC773918, KC773922–773952, KC773958–KC773984, GU270494–GU270550, HM061083–HM061092. The 454 pyrosequencing data are deposited at the DNA Data Bank of Japan (DDJB) under Accession Numbers DRA001139 and DRA001141 for 16S rRNA and 18S rRNA genes, respectively.

## Supporting Information

**Figure S1.** Geographic locations of the experimental sites. The work involved three local soils from two Experimental Research Stations: Chao soil in Fengqiu (FQ); purple and red soils in Yingtan (YT). In the soil transplantation experiment, purple and red soils were placed in parallel in two geographic locations (i.e., FQ and YT). Of note, the monthly average of temperature ranges from −1.0°C to 26.7°C in FQ and 5.1°C to 29.5°C in YT. After 20 years, a total of four soil samples were taken from the transplanted soils at two sites and subjected to chemical, physical, and microbiological analysis. Additionally, the local Chao soil in FQ was also included in this work as a control. **Figure S2.** Orientation plots generated by canonical correlation analysis of bacterial communities as estimated by 16S rDNA DGGE analysis. Environmental variables were subjected to the forward selection procedure using the Monte Carlo permutation test (P<0.05) and variance inflation factors (VIF<20). Variation in the bacterial communities can be explained by the factor of soil pH, water content, total N, and SOC at the level of 11.2%, 9.3%, 7.3%, and 6.0%, respectively. Soil samples are designated by location (FQ, Fengqiu; YT, Yingtan) and soil type (Chao, purple or red soil). **Figure S3.** Rarefaction curves of the total number of OTUs against the total number of reads for the 16S rDNA (A) and 18S rDNA (B) sequences. Operational taxonomic units are defined by the 97% similarity cutoff. Soil samples are designated by location (FQ, Fengqiu; YT, Yingtan) and soil type (Chao, purple or red soil). **Figure S4.** Cluster analysis of operational taxonomic units (OTUs) belonging to the four major types of microbial eukaryotes. Phylogenic distance was estimated using the 18S rDNA pyrosequencing data. OTUs are defined at the level of 97% sequence similarity and their relative abundance is indicated with different colors. Soil samples are designated by location (FQ, Fengqiu; YT, Yingtan) and soil type (Chao, purple, or red soils).

**Table S1.** Abundance of dominant bacterial genera identified by 16S rDNA 454 pyrosequencing. **Table S2.** Abundances of eukaryotic families identified in the 18S rDNA 454 pyrosequencing. **Table S3.** List of oligonucleotide primers used in this study.
