# Supplementary material for: A long-term field experiment of soil transplantation demonstrating the role of contemporary geographic separation in shaping soil microbial community structure
Source: Ecol Evol. 2014 Mar 6;4(7):1073–87. doi: 10.1002/ece3.1006 (PMC3997323; doi:10.1002/ece3.1006)
Supplement: Supplementary file 1 — Figure S1. Geographic locations of the experimental sites. The work involved three local soils from two Experimental Research Stations: Chao soil in Fengqiu (FQ); purple and red soils in Yingtan (YT). In the soil transplantation experiment, purple and red soils were placed in parallel in two geographic locations (i.e., FQ and YT). Of note, the monthly average of temperature ranges from −1.0°C to 26.7°C in FQ and 5.1°C to 29.5°C in YT. After 20 years, a total of four soil samples were taken from the transplanted soils at two sites and subjected to chemical, physical, and microbiological analysis. Additionally, the local Chao soil in FQ was also included in this work as a control. Figure S2. Orientation plots generated by canonical correlation analysis of bacterial communities as estimated by 16S rDNA DGGE analysis. Environmental variables were subjected to the forward selection procedure using the Monte Carlo permutation test (P<0.05) and variance inflation factors (VIF<20). Variation in the bacterial communities can be explained by the factor of soil pH, water content, total N, and SOC at the level of 11.2%, 9.3%, 7.3%, and 6.0%, respectively. Soil samples are designated by location (FQ, Fengqiu; YT, Yingtan) and soil type (Chao, purple or red soil). Figure S3. Rarefaction curves of the total number of OTUs against the total number of reads for the 16S rDNA (A) and 18S rDNA (B) sequences. Operational taxonomic units are defined by the 97% similarity cutoff. Soil samples are designated by location (FQ, Fengqiu; YT, Yingtan) and soil type (Chao, purple or red soil). Figure S4. Cluster analysis of operational taxonomic units (OTUs) belonging to the four major types of microbial eukaryotes. Phylogenic distance was estimated using the 18S rDNA pyrosequencing data. OTUs are defined at the level of 97% sequence similarity and their relative abundance is indicated with different colors. Soil samples are designated by location (FQ, Fengqiu; YT, Yingtan) and soil type (Chao, [file ece30004-1073-sd1.pdf]

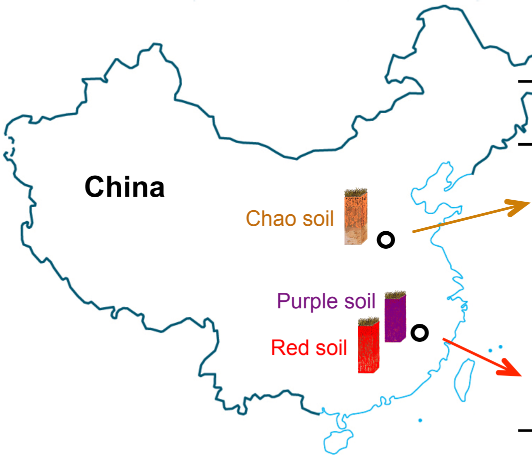

Site	Geographic location	Temperature	Precipitation
FQ	114°24’ E, 35°00’ N (warm temperate zone)	13.9 °C	605 mm
YT	116°55’E, 28°13’N (mid-subtropical zone)	17.6 °C	1795 mm

Figure S1. Geographic locations of the experimental sites. The work involved three local soils from two Experimental Research Stations: Chao soil in Fengqiu (FQ); purple and red soils in Yingtan (YT). In the soil transplantation experiment purple and red soils were placed in parallel in two geographic locations (i.e., FQ and YT). Of note, the monthly average of temperature ranges from -1.0 °C to 26.7 °C in FQ, and 5.1 °C to 29.5 °C in YT. After 20 years, a total of 4 soil samples were taken from the transplanted soils at two sites, and subjected to chemical, physical and microbiological analysis. Additionally, the local Chao soil in FQ was also included in this work as a control.

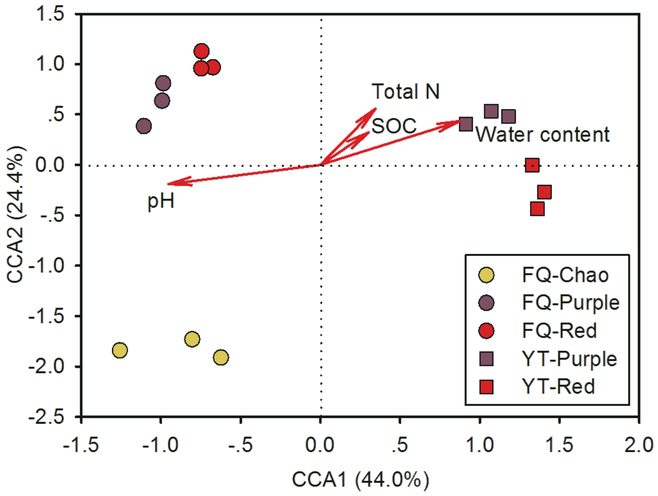

Fig. S2. Orientation plots generated by CCA analysis of bacterial communities as estimated by 16S rDNA DGGE analysis. Environmental variables were subjected to the forward selection procedure using the Monte Carlo permutation test ($P < 0.05$) and variance inflation factors (VIF < 20). Variation of the bacterial communities can be explained by the factor of soil pH, water content, total N and SOC at the level of 11.2%, 9.3%, 7.3% and 6.0%, respectively. Soil samples are designated by location (FQ, Fengqiu; YT, Yingtan) and soil type (Chao, purple or red soil).

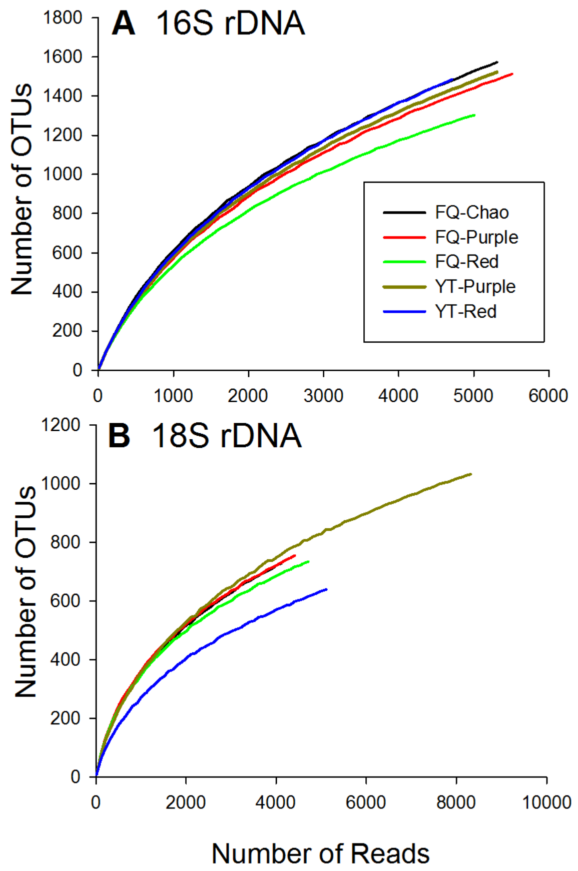

Figure S3. Rarefaction curves of the total number of OUTs against the total number of reads for the 16S rDNA (A) and 18S rDNA (B) sequences. OTUs are defined by the 97% similarity cutoff. Soil samples are designated by location (FQ, Fengqiu; YT, Yingtan) and soil type (Chao, Purple or Red soil).

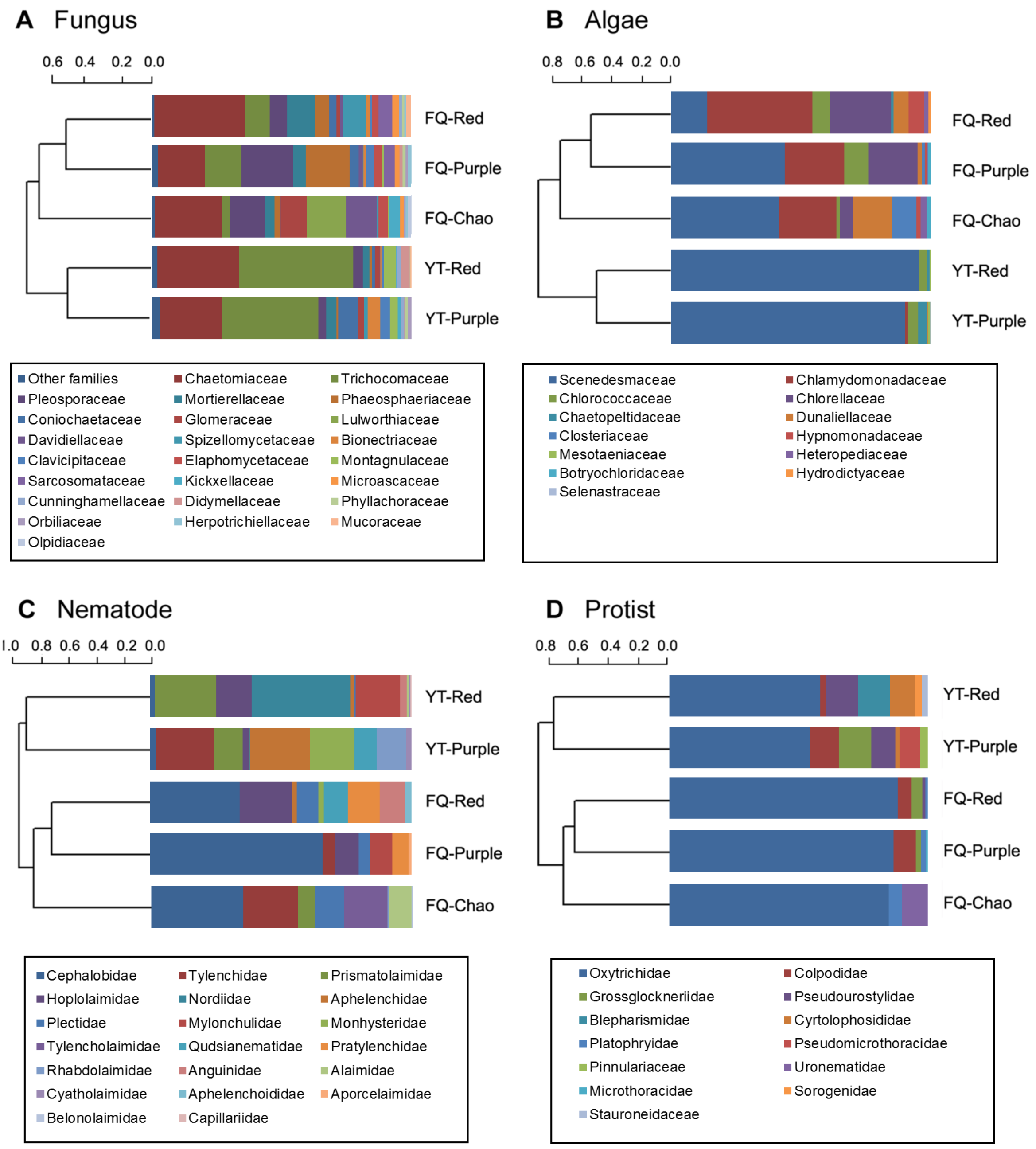

Fig. S4. Cluster analysis of OTUs belonging to the four major types of microbial eukaryotes. Phylogenic distance was estimated using the 18S rDNA pyrosequencing data. OTUs are defined at the level of 97% sequence similarity and their relative abundance is indicated with different colors. Soil samples are designated by location (FQ, Fengqiu; YT, Yingtan) and soil type (Chao, purple or red soils).
