# Supplementary material for: A long-term field experiment of soil transplantation demonstrating the role of contemporary geographic separation in shaping soil microbial community structure
Source: Ecol Evol. 2014 Mar 6;4(7):1073–87. doi: 10.1002/ece3.1006 (PMC3997323; doi:10.1002/ece3.1006)
Supplement: Supplementary file 2 — Table S1. Abundance of dominant bacterial genera identified by 16S rDNA 454 pyrosequencing. Table S2. Abundances of eukaryotic families identified in the 18S rDNA 454 pyrosequencing. Table S3. List of oligonucleotide primers used in this study. [file ece30004-1073-sd2.docx]

**Table S1** Abundance of dominant bacterial genera identified by 16S rDNA 454 pyrosequencing

| Groups | FQ-Chao | FQ-Purple | FQ-Red | YT-Purple | YT-Red |
| --- | --- | --- | --- | --- | --- |
| *Bacillus* | 140 | 50 | 33 | 271 | 115 |
| *Sphingomonas* | 36 | 192 | 145 | 34 | 35 |
| *Nocardioides* | 79 | 134 | 169 | 21 | 7 |
| *Arthrobacter* | 45 | 68 | 207 | 9 | 3 |
| *Streptomyces* | 21 | 45 | 53 | 75 | 62 |
| *Marmoricola* | 64 | 57 | 103 | 10 | 2 |
| *Roseiflexus* | 16 | 20 | 43 | 118 | 35 |
| *Bradyrhizobium* | 23 | 56 | 32 | 41 | 76 |
| *Mycobacterium* | 16 | 48 | 67 | 50 | 40 |
| *Flexibacter* | 46 | 92 | 53 | 1 | 0 |
| *Nitrospira* | 70 | 17 | 30 | 24 | 26 |
| *Skermanella* | 35 | 68 | 55 | 8 | 0 |
| *Haliangium* | 38 | 26 | 16 | 54 | 15 |
| *Nitrosospira* | 57 | 40 | 47 | 2 | 1 |
| *Agromyces* | 29 | 29 | 84 | 1 | 0 |
| *Solirubrobacter* | 38 | 41 | 34 | 14 | 9 |
| *Gemmatimonas* | 2 | 36 | 20 | 21 | 42 |
| *Devosia* | 26 | 55 | 23 | 13 | 0 |
| *Steroidobacter* | 64 | 20 | 28 | 4 | 0 |
| *Candidatus Solibacter* | 11 | 1 | 4 | 23 | 69 |
| *Acidothermus* | 0 | 0 | 0 | 22 | 82 |
| *Flavisolibacter* | 14 | 28 | 49 | 1 | 11 |
| *Rhodobium* | 44 | 22 | 6 | 29 | 2 |
| *Alicyclobacillus* | 23 | 15 | 17 | 15 | 29 |
| *Pedomicrobium* | 43 | 15 | 4 | 30 | 5 |
| *Chloracidobacterium* | 23 | 36 | 29 | 6 | 1 |
| *Mesorhizobium* | 12 | 17 | 57 | 6 | 0 |
| *Microbacterium* | 11 | 26 | 55 | 0 | 0 |
| *Paenibacillus* | 13 | 12 | 15 | 27 | 15 |
| *Gemmata* | 4 | 12 | 31 | 9 | 12 |
| *Hyphomicrobium* | 42 | 6 | 15 | 0 | 2 |
| *Clostridium* | 23 | 7 | 6 | 21 | 7 |
| *Anaeromyxobacter* | 9 | 3 | 6 | 37 | 8 |
| *Candidatus Alysiosphaera* | 25 | 17 | 10 | 7 | 3 |
| *Micromonospora* | 23 | 11 | 14 | 8 | 5 |
| *Nordella* | 19 | 5 | 13 | 22 | 2 |
| *Rhizobium* | 7 | 37 | 15 | 0 | 0 |
| *Leptolyngbya* | 0 | 51 | 1 | 3 | 1 |
| *Lysobacter* | 12 | 20 | 11 | 8 | 3 |
| *Pirellula* | 27 | 2 | 14 | 3 | 7 |
| *Bryobacter* | 0 | 5 | 18 | 10 | 19 |
| *Rhodococcus* | 32 | 4 | 10 | 3 | 3 |
| *Rubellimicrobium* | 10 | 30 | 12 | 0 | 0 |
| *Sorangium* | 4 | 5 | 8 | 14 | 20 |
| *Aminobacter* | 11 | 25 | 13 | 1 | 0 |
| *Sporosarcina* | 19 | 17 | 6 | 1 | 3 |
| *Ilumatobacter* | 34 | 9 | 0 | 1 | 1 |
| *Aeromicrobium* | 4 | 3 | 36 | 0 | 0 |
| *Zavarzinella* | 2 | 4 | 7 | 12 | 18 |
| *Burkholderia* | 0 | 0 | 0 | 9 | 31 |
| *Pseudonocardia* | 5 | 10 | 8 | 10 | 7 |
| *Microcoleus* | 0 | 21 | 18 | 0 | 0 |
| *Geodermatophilus* | 1 | 14 | 17 | 4 | 2 |
| *Paucimonas* | 0 | 34 | 0 | 3 | 0 |
| *Ramlibacter* | 2 | 19 | 13 | 3 | 0 |
| *Candidatus Entotheonella* | 14 | 5 | 1 | 12 | 3 |
| *Kribbella* | 8 | 6 | 11 | 2 | 8 |
| *Phenylobacterium* | 9 | 7 | 6 | 4 | 9 |
| *Pedobacter* | 9 | 8 | 16 | 0 | 0 |
| *Planktothrix* | 0 | 30 | 3 | 0 | 0 |
| *Aquicella* | 10 | 9 | 5 | 7 | 1 |
| *Patulibacter* | 0 | 3 | 5 | 17 | 7 |
| *Pseudolabrys* | 10 | 7 | 6 | 2 | 7 |
| *Caldilinea* | 10 | 5 | 5 | 5 | 5 |
| *Massilia* | 7 | 1 | 10 | 1 | 10 |
| *Opitutus* | 1 | 21 | 5 | 2 | 0 |
| *Rhodanobacter* | 0 | 9 | 13 | 3 | 4 |
| *Xanthomonas* | 4 | 12 | 11 | 0 | 0 |
| *Candidatus Koribacter* | 0 | 1 | 0 | 14 | 11 |
| *Planctomyces* | 8 | 1 | 8 | 7 | 2 |
| *Blastococcus* | 15 | 6 | 4 | 0 | 0 |
| *Pontibacter* | 3 | 17 | 5 | 0 | 0 |
| *Iamia* | 16 | 4 | 3 | 0 | 0 |
| *Singulisphaera* | 0 | 0 | 4 | 8 | 11 |
| *Frankia* | 0 | 2 | 0 | 4 | 16 |
| *Thermomonas* | 2 | 6 | 14 | 0 | 0 |
| *Microvirga* | 10 | 3 | 6 | 0 | 1 |
| *Nitrosomonas* | 13 | 3 | 4 | 0 | 0 |
| *Rhodoplanes* | 0 | 3 | 0 | 14 | 3 |
| *Arenimonas* | 1 | 11 | 0 | 6 | 1 |
| *Lysinibacillus* | 3 | 0 | 0 | 16 | 0 |
| *Vampirovibrio* | 0 | 1 | 0 | 15 | 3 |
| *Nannocystis* | 2 | 10 | 6 | 0 | 0 |
| *Caulobacter* | 1 | 13 | 1 | 2 | 0 |
| *Legionella* | 0 | 3 | 6 | 7 | 0 |
| *Blastopirellula* | 15 | 0 | 0 | 0 | 0 |
| *Rhodocytophaga* | 3 | 9 | 3 | 0 | 0 |
| *Amycolatopsis* | 2 | 9 | 1 | 2 | 0 |
| *Methylobacterium* | 0 | 1 | 0 | 12 | 1 |
| *Paracoccus* | 4 | 8 | 2 | 0 | 0 |
| *Sphaerobacter* | 0 | 11 | 3 | 0 | 0 |
| *Variovorax* | 3 | 6 | 4 | 0 | 1 |
| *Achromobacter* | 0 | 0 | 2 | 5 | 6 |
| *Acidobacterium* | 0 | 0 | 0 | 2 | 11 |
| *Chthoniobacter* | 1 | 0 | 3 | 9 | 0 |
| *Geminicoccus* | 0 | 6 | 7 | 0 | 0 |
| *Herpetosiphon* | 0 | 9 | 4 | 0 | 0 |
| *Microlunatus* | 0 | 6 | 6 | 1 | 0 |
| *Niastella* | 2 | 0 | 4 | 7 | 0 |
| *Paenisporosarcina* | 2 | 11 | 0 | 0 | 0 |
| *Pseudomonas* | 2 | 10 | 0 | 1 | 0 |
| *Crossiella* | 0 | 0 | 0 | 0 | 12 |
| *Rhizobacter* | 0 | 3 | 8 | 1 | 0 |
| *Acidisoma* | 0 | 0 | 0 | 1 | 10 |
| *Streptosporangium* | 0 | 4 | 7 | 0 | 0 |
| *Brevundimonas* | 0 | 8 | 2 | 0 | 0 |
| *Derxia* | 0 | 2 | 8 | 0 | 0 |
| *Herbaspirillum* | 0 | 4 | 6 | 0 | 0 |
| *Lutispora* | 5 | 0 | 0 | 5 | 0 |
| *Pseudospirillum* | 0 | 1 | 9 | 0 | 0 |
| *Actinoallomurus* | 0 | 0 | 2 | 2 | 5 |
| *Bosea* | 0 | 2 | 7 | 0 | 0 |
| *Chryseobacterium* | 0 | 1 | 8 | 0 | 0 |
| *Phormidium* | 0 | 9 | 0 | 0 | 0 |
| *Planosporangium* | 1 | 0 | 3 | 0 | 5 |
| *Rhodomicrobium* | 7 | 0 | 0 | 2 | 0 |
| *Roseomonas* | 2 | 5 | 2 | 0 | 0 |
| *Sporacetigenium* | 6 | 2 | 1 | 0 | 0 |
| *Cohnella* | 3 | 5 | 0 | 0 | 0 |
| *Comamonas* | 0 | 8 | 0 | 0 | 0 |
| *Cupriavidus* | 2 | 0 | 0 | 4 | 2 |
| *Jiangella* | 8 | 0 | 0 | 0 | 0 |
| *Luteimonas* | 0 | 0 | 8 | 0 | 0 |
| *Brevibacillus* | 0 | 0 | 0 | 5 | 2 |
| *Euzebya* | 5 | 1 | 1 | 0 | 0 |
| *Geobacter* | 5 | 0 | 1 | 1 | 0 |
| *Nocardia* | 0 | 1 | 0 | 5 | 1 |
| *Rhodopirellula* | 0 | 7 | 0 | 0 | 0 |
| *Sporocytophaga* | 0 | 0 | 7 | 0 | 0 |
| *Acidovorax* | 0 | 3 | 3 | 0 | 0 |
| *Caenimonas* | 1 | 0 | 1 | 3 | 1 |
| *Dokdonella* | 0 | 0 | 0 | 6 | 0 |
| *Flavobacterium* | 2 | 4 | 0 | 0 | 0 |
| *Limnobacter* | 5 | 1 | 0 | 0 | 0 |
| *Nubsella* | 0 | 6 | 0 | 0 | 0 |
| *Phaselicystis* | 4 | 0 | 1 | 1 | 0 |
| *Pseudoxanthomonas* | 0 | 6 | 0 | 0 | 0 |
| *Symbiobacterium* | 0 | 1 | 5 | 0 | 0 |
| *Adhaeribacter* | 1 | 4 | 0 | 0 | 0 |
| *Azohydromonas* | 5 | 0 | 0 | 0 | 0 |
| *Bacteriovorax* | 0 | 5 | 0 | 0 | 0 |
| *Bdellovibrio* | 1 | 4 | 0 | 0 | 0 |
| *Cystobacter* | 0 | 1 | 4 | 0 | 0 |
| *Ideonella* | 0 | 0 | 0 | 5 | 0 |
| *Kineococcus* | 3 | 2 | 0 | 0 | 0 |
| *Ornithinimicrobium* | 0 | 0 | 5 | 0 | 0 |
| *Planomicrobium* | 5 | 0 | 0 | 0 | 0 |
| *Pusillimonas* | 0 | 0 | 5 | 0 | 0 |
| *Sphingobacterium* | 0 | 5 | 0 | 0 | 0 |
| *Terrabacter* | 0 | 0 | 2 | 3 | 0 |
| *Conexibacter* | 0 | 0 | 0 | 4 | 0 |
| *Kibdelosporangium* | 0 | 0 | 0 | 1 | 3 |
| *Thermosporothrix* | 0 | 0 | 0 | 0 | 4 |
| *Candidatus Xiphinematobacter* | 0 | 1 | 0 | 0 | 2 |
| *Chitinophaga* | 1 | 1 | 1 | 0 | 0 |
| *Dyella* | 0 | 0 | 0 | 0 | 3 |
| *Hymenobacter* | 0 | 0 | 3 | 0 | 0 |
| *Nitrobacter* | 0 | 3 | 0 | 0 | 0 |
| *Polaromonas* | 0 | 3 | 0 | 0 | 0 |
| *Shimazuella* | 0 | 2 | 1 | 0 | 0 |
| *Sinomonas* | 0 | 0 | 0 | 1 | 2 |
| *Terrimonas* | 0 | 2 | 1 | 0 | 0 |
| *Actinocorallia* | 2 | 0 | 0 | 0 | 0 |
| *Actinomadura* | 0 | 2 | 0 | 0 | 0 |
| *Albidiferax* | 0 | 0 | 2 | 0 | 0 |
| *Asteroleplasma* | 2 | 0 | 0 | 0 | 0 |
| *Byssovorax* | 0 | 0 | 0 | 0 | 2 |
| *Candidatus Odyssella* | 0 | 0 | 2 | 0 | 0 |
| *Chloroflexus* | 0 | 2 | 0 | 0 | 0 |
| *Cloacibacterium* | 0 | 2 | 0 | 0 | 0 |
| *Dactylosporangium* | 0 | 0 | 0 | 2 | 0 |
| *Edaphobacter* | 0 | 0 | 0 | 1 | 1 |
| *Hydrogenophaga* | 0 | 2 | 0 | 0 | 0 |
| *Kineosporia* | 0 | 0 | 0 | 2 | 0 |
| *Ktedonobacter* | 0 | 0 | 0 | 2 | 0 |
| *Luedemannella* | 0 | 0 | 1 | 1 | 0 |
| *Methylibium* | 0 | 1 | 1 | 0 | 0 |
| *Nakamurella* | 0 | 2 | 0 | 0 | 0 |
| *Oxobacter* | 0 | 1 | 1 | 0 | 0 |
| *Porphyrobacter* | 0 | 2 | 0 | 0 | 0 |
| *Rhodobacter* | 0 | 2 | 0 | 0 | 0 |
| *Sandaracinobacter* | 0 | 2 | 0 | 0 | 0 |
| *Sporomusa* | 0 | 0 | 0 | 2 | 0 |
| *Actinoplanes* | 0 | 0 | 1 | 0 | 0 |
| *Alkaliphilus* | 0 | 1 | 0 | 0 | 0 |
| *Anaerolinea* | 0 | 1 | 0 | 0 | 0 |
| *Anoxybacillus* | 0 | 1 | 0 | 0 | 0 |
| *Azospira* | 0 | 0 | 0 | 0 | 1 |
| *Balneimonas* | 0 | 0 | 0 | 1 | 0 |
| *Candidatus Nitrotoga* | 1 | 0 | 0 | 0 | 0 |
| *Cellvibrio* | 0 | 1 | 0 | 0 | 0 |
| *Defluviicoccus* | 0 | 1 | 0 | 0 | 0 |
| *Desulfosporosinus* | 0 | 0 | 1 | 0 | 0 |
| *Dyadobacter* | 0 | 1 | 0 | 0 | 0 |
| *Enhygromyxa* | 0 | 1 | 0 | 0 | 0 |
| *Hirschia* | 0 | 1 | 0 | 0 | 0 |
| *Inquilinus* | 0 | 1 | 0 | 0 | 0 |
| *Isosphaera* | 0 | 0 | 0 | 0 | 1 |
| *Lacibacter* | 0 | 1 | 0 | 0 | 0 |
| *Marinibacillus* | 0 | 0 | 0 | 0 | 1 |
| *Marinicella* | 1 | 0 | 0 | 0 | 0 |
| *Phyllobacterium* | 0 | 1 | 0 | 0 | 0 |
| *Propionivibrio* | 1 | 0 | 0 | 0 | 0 |
| *Rathayibacter* | 1 | 0 | 0 | 0 | 0 |
| *Rubrobacter* | 0 | 0 | 1 | 0 | 0 |
| *Rummeliibacillus* | 0 | 0 | 0 | 0 | 1 |
| *Sphingobium* | 0 | 1 | 0 | 0 | 0 |
| *Streptococcus* | 0 | 0 | 0 | 1 | 0 |
| *Thermincola* | 1 | 0 | 0 | 0 | 0 |
| *Truepera* | 0 | 1 | 0 | 0 | 0 |

**Table S2** Abundances of eukaryotic families identified in the 18S rDNA 454 pyrosequencing

| Groups | FQ-Chao | FQ-Purple | FQ-Red | YT-Purple | YT-Red |
| --- | --- | --- | --- | --- | --- |
| Fungi |  |  |  |  |  |
| *Chaetomiaceae* | 271 | 144 | 326 | 341 | 255 |
| *Trichocomaceae* | 34 | 111 | 89 | 523 | 356 |
| *Pleosporaceae* | 140 | 157 | 62 | 43 | 31 |
| *Mortierellaceae* | 40 | 40 | 102 | 56 | 19 |
| *Phaeosphaeriaceae* | 21 | 133 | 49 | 12 | 9 |
| *Coniochaetaceae* | 4 | 27 | 27 | 106 | 9 |
| *Glomeraceae* | 107 | 0 | 13 | 33 | 14 |
| *Lulworthiaceae* | 157 | 0 | 0 | 0 | 0 |
| *Davidiellaceae* | 126 | 14 | 11 | 1 | 3 |
| *Spizellomycetaceae* | 3 | 2 | 81 | 20 | 0 |
| *Clavicipitaceae* | 4 | 26 | 7 | 51 | 7 |
| *Bionectriaceae* | 0 | 6 | 15 | 70 | 4 |
| *Elaphomycetaceae* | 39 | 24 | 23 | 0 | 0 |
| *Montagnulaceae* | 1 | 5 | 0 | 43 | 37 |
| *Sarcosomataceae* | 1 | 34 | 50 | 0 | 0 |
| *Kickxellaceae* | 47 | 0 | 0 | 19 | 3 |
| *Microascaceae* | 13 | 15 | 23 | 1 | 0 |
| *Cunninghamellaceae* | 1 | 0 | 11 | 15 | 14 |
| *Didymellaceae* | 0 | 8 | 1 | 5 | 26 |
| *Phyllachoraceae* | 0 | 9 | 9 | 16 | 3 |
| *Orbiliaceae* | 7 | 9 | 0 | 19 | 0 |
| *Herpotrichiellaceae* | 10 | 10 | 5 | 0 | 0 |
| *Mucoraceae* | 0 | 0 | 20 | 0 | 3 |
| *Olpidiaceae* | 14 | 0 | 0 | 0 | 0 |
| *Dipodascaceae* | 0 | 7 | 0 | 5 | 0 |
| *Diversisporaceae* | 0 | 0 | 0 | 7 | 4 |
| *Pezizaceae* | 0 | 0 | 0 | 10 | 0 |
| *Sclerotiniaceae* | 5 | 0 | 4 | 0 | 1 |
| *Apiosporaceae* | 1 | 1 | 2 | 1 | 3 |
| *Eustigmataceae* | 4 | 0 | 0 | 1 | 0 |
| *Verrucariaceae* | 0 | 0 | 0 | 0 | 5 |
| *Endogonaceae* | 0 | 0 | 0 | 5 | 0 |
| *Cochlonemataceae* | 0 | 3 | 1 | 0 | 0 |
| *Rhizophydiaceae* | 1 | 2 | 1 | 0 | 0 |
| *Megachytriaceae* | 0 | 0 | 0 | 4 | 0 |
| *Botryosphaeriaceae* | 0 | 0 | 0 | 4 | 0 |
| *Hypocreaceae* | 0 | 0 | 0 | 3 | 0 |
| *Ustilaginaceae* | 0 | 3 | 0 | 0 | 0 |
| *Corticiaceae* | 0 | 0 | 0 | 3 | 0 |
| *Blastocladiaceae* | 1 | 1 | 0 | 0 | 0 |
| *Basidiobolaceae* | 0 | 0 | 0 | 0 | 2 |
| *Ascobolaceae* | 1 | 1 | 0 | 0 | 0 |
| *Saccharomycetaceae* | 0 | 1 | 0 | 1 | 0 |
| *Lipomycetaceae* | 0 | 0 | 0 | 0 | 1 |
| *Glomerellaceae* | 0 | 0 | 0 | 0 | 1 |
| *Sporormiaceae* | 0 | 0 | 0 | 0 | 1 |
| *Pyronemataceae* | 0 | 0 | 0 | 1 | 0 |
| *Ascodesmidaceae* | 0 | 0 | 0 | 1 | 0 |
| *Nectriaceae* | 1 | 0 | 0 | 0 | 0 |
| *Catenariaceae* | 0 | 0 | 1 | 0 | 0 |
| *Myxotrichaceae* | 0 | 0 | 1 | 0 | 0 |
| Nematode |  |  |  |  |  |
| *Cephalobidae* | 95 | 125 | 71 | 6 | 5 |
| *Tylenchidae* | 56 | 9 | 0 | 55 | 0 |
| *Prismatolaimidae* | 18 | 0 | 0 | 27 | 54 |
| *Hoplolaimidae* | 0 | 17 | 41 | 6 | 31 |
| *Nordiidae* | 0 | 0 | 0 | 1 | 87 |
| *Aphelenchidae* | 0 | 0 | 4 | 57 | 3 |
| *Plectidae* | 30 | 8 | 17 | 0 | 2 |
| *Mylonchulidae* | 0 | 16 | 0 | 0 | 39 |
| *Monhysteridae* | 0 | 0 | 4 | 42 | 0 |
| *Tylencholaimidae* | 44 | 0 | 0 | 0 | 0 |
| *Qudsianematidae* | 0 | 0 | 19 | 21 | 0 |
| *Pratylenchidae* | 0 | 12 | 25 | 0 | 0 |
| *Rhabdolaimidae* | 2 | 0 | 0 | 28 | 0 |
| *Anguinidae* | 0 | 0 | 20 | 0 | 6 |
| *Alaimidae* | 23 | 0 | 0 | 0 | 2 |
| *Cyatholaimidae* | 0 | 0 | 0 | 5 | 1 |
| *Aphelenchoididae* | 0 | 0 | 5 | 0 | 0 |
| *Aporcelaimidae* | 0 | 2 | 0 | 0 | 0 |
| *Belonolaimidae* | 1 | 0 | 0 | 0 | 0 |
| *Capillariidae* | 0 | 0 | 0 | 0 | 1 |
| Protist |  |  |  |  |  |
| *Oxytrichidae* | 17 | 132 | 84 | 35 | 24 |
| *Colpodidae* | 0 | 13 | 5 | 7 | 1 |
| *Grossglockneriidae* | 0 | 3 | 4 | 8 | 0 |
| *Pseudourostylidae* | 0 | 0 | 1 | 6 | 5 |
| *Pseudomicrothoracidae* | 0 | 0 | 0 | 5 | 0 |
| *Platophryidae* | 1 | 3 | 1 | 0 | 0 |
| *Cyrtolophosididae* | 0 | 0 | 0 | 1 | 4 |
| *Blepharismidae* | 0 | 0 | 0 | 0 | 5 |
| *Uronematidae* | 2 | 0 | 0 | 0 | 0 |
| *Pinnulariaceae* | 0 | 0 | 0 | 2 | 0 |
| *Sorogenidae* | 0 | 0 | 0 | 0 | 1 |
| *Microthoracidae* | 0 | 1 | 0 | 0 | 0 |
| *Stauroneidaceae* | 0 | 0 | 0 | 0 | 1 |
| Alga |  |  |  |  |  |
| *Scenedesmaceae* | 53 | 81 | 17 | 777 | 414 |
| *Chlamydomonadaceae* | 28 | 42 | 48 | 10 | 1 |
| *Chlorococcaceae* | 2 | 17 | 8 | 34 | 13 |
| *Chlorellaceae* | 6 | 35 | 28 | 0 | 0 |
| *Chaetopeltidaceae* | 0 | 0 | 1 | 30 | 4 |
| *Dunaliellaceae* | 19 | 3 | 7 | 2 | 0 |
| *Closteriaceae* | 12 | 2 | 0 | 0 | 0 |
| *Mesotaeniaceae* | 0 | 0 | 0 | 8 | 2 |
| *Hypnomonadaceae* | 2 | 1 | 7 | 0 | 0 |
| *Heteropediaceae* | 3 | 1 | 2 | 0 | 0 |
| *Botryochloridaceae* | 2 | 2 | 0 | 0 | 0 |
| *Selenastraceae* | 0 | 0 | 0 | 1 | 0 |
| *Hydrodictyaceae* | 0 | 0 | 1 | 0 | 0 |

**Table S3** List of oligonucleotide primers used in this study

| Application | Primer | Sequence (5’ to 3’) | References |
| --- | --- | --- | --- |
| 16S rDNA  DGGE | P2  P3^a^ | ATTACCGCGGCTGCTGG  CCTACGGGAGGCAGCAG^a^ | ([Muyzer *et al.* 1993](#_ENREF_46)) |
| Archeael *amoA* | Arch-amoAF  Arch-amoAR | STAATGGTCTGGCTTAGACG  GCGGCCATCCATCTGTATGT | ([Francis 2005](#_ENREF_14)) |
| Bacterial *amoA* | amoA-1F  amoA-2R | GGGGTTTCTACTGGTGGT  CCCCTCKGSAAAGCCTTCTTC | ([Rotthauwe *et al.* 1997](#_ENREF_60)) |
| *NifH* | nifH-F  nifH-R | AAAGGYGGWATCGGYAARTCCACCAC  TTGTTSGCSGCRTACATSGCCATCAT | ([Rosch *et al.* 2002](#_ENREF_59)) |
| *NirK* | nirK-1F  nirK-5R | GGMATGGTBCCSTGGCA  GCCTCGATCAGRTTRTGG | ([Braker *et al.* 1998](#_ENREF_5)) |
| 16S rDNA  pyrosequencing | 8f  533r | AGAGTTTGATCCTGGCTCAG  TTACCGCGGCTGCTGGCAC | ([Lu *et al.* 2012](#_ENREF_39)) |
| 18S rDNA  pyrosequencing | 3NDf  V4-R2 | GGCAAGTCTGGTGCCAG  ACGGTATCT(AG)ATC(AG)TCTTCG | ([Brate *et al.* 2010](#_ENREF_6)) |

^a^A 40-nucleotide GC-rich sequence (GC clamp) is incorporated into the 5’-end of P3.
